# Modulation of LAT1 (SLC7A5) transporter activity and stability by membrane cholesterol

**DOI:** 10.1038/srep43580

**Published:** 2017-03-08

**Authors:** David Dickens, George N. Chiduza, Gareth S. A. Wright, Munir Pirmohamed, Svetlana V. Antonyuk, S. Samar Hasnain

**Affiliations:** 1 Department of Molecular and Clinical Pharmacology, University of Liverpool, Liverpool, UK; 2 Molecular Biophysics Group, Institute of Integrative Biology, Faculty of Health and Life Sciences, University of Liverpool, UK

## Abstract

LAT1 (SLC7A5) is a transporter for both the uptake of large neutral amino acids and a number of pharmaceutical drugs. It is expressed in numerous cell types including T-cells, cancer cells and brain endothelial cells. However, mechanistic knowledge of how it functions and its interactions with lipids are unknown or limited due to inability of obtaining stable purified protein in sufficient quantities. Our data show that depleting cellular cholesterol reduced the V_max_ but not the K_m_ of the LAT1 mediated uptake of a model substrate into cells (L-DOPA). A soluble cholesterol analogue was required for the stable purification of the LAT1 with its chaperon CD98 (4F2hc,SLC3A2) and that this stabilised complex retained the ability to interact with a substrate. We propose cholesterol interacts with the conserved regions in the LAT1 transporter that have been shown to bind to cholesterol/CHS in *Drosophila melanogaster* dopamine transporter. In conclusion, LAT1 is modulated by cholesterol impacting on its stability and transporter activity. This novel finding has implications for other SLC7 family members and additional eukaryotic transporters that contain the LeuT fold.

The L-Type Amino Acid Transporter 1 (LAT1,SLC7A5) is part of the SLC7 family and forms a heterodimer with CD98 via a disulphide bond^1^. CD98 (4F2hc,SLC3A2) is a type II glycoprotein that functions as a chaperone for LAT1, stabilising and facilitating its translocation to the plasma membrane. LAT1 is the functional unit of the complex^2^ and substrates include a range of large neutral amino acids such as tyrosine, leucine, isoleucine, valine and phenylalanine as well as pharmaceutical drugs including L-DOPA and gabapentin^3^,^4^.

LAT1 is expressed in many tissues of the body, functioning as a sodium independent antiporter with a 1:1 stoichiometry^5^. LAT1 is highly expressed in brain endothelial cells, in the blood-brain barrier and in the inner blood retinal barrier^6^,^7^. LAT1 is a key transporter in the uptake of substrates into the brain and has been proposed as a target for enhanced delivery into the brain for new molecular entities^8^. LAT1 expression is also observed at the placenta suggesting a role for providing the essential amino acids needed for the growing foetus^9^. The global knockout of LAT1 in mice has been found to be embryonically lethal which could be due to its transport role at the placenta or because it is essential for cells in terms of the uptake of large neutral amino acids or both^10^. A conditional knock out of LAT1 in T-cells of mice has shown that it is the main L-type amino acid transporter in this cell type and is required for the metabolic reprogramming essential for T-cell differentiation^11^.

In many human tumours, LAT1 is highly overexpressed which is thought to play an important role in tumour growth and disease progression^12^. LAT1 has thus been proposed as a novel target for cancer treatment. An example of this approach is the generation of a high affinity LAT1 inhibitor of (JPH203/ KYT-0353), that inhibits tumour growth *in vivo* and is currently undergoing a Phase I clinical trial in humans (UMIN000016546) as a novel adjuvant treatment approach for solid tumours^13^.

The mechanistic knowledge of how LAT1 functions as a transporter is based on our knowledge of the LeuT fold prokaryotic transporters of known structure despite their low sequence identity to LAT1 (<21%)^14^,^15^. From these low similarity homologues a predictive structure of LAT1 was generated and docked with a chemical library *in silico* which identified additional ligands^16^,^17^. This approach has proposed that LAT1 can transport substrates by the alternative-access mechanism where transporters undergo several conformational changes to translocate the substrate across the plasma membrane^3^,^16^,^17^. The CD98 ectodomain structure has been solved by x-ray crystallography^18^ but does not provide an insight into how the interaction between the two components of the heterodimer affect the transport cycle of LAT1. LAT2 (SLC7A8), which has 52.8% identity to LAT1, is stabilised by CD98 surrounding the extracellular regions of the transporter^19^.

Cholesterol is an important component of the plasma membrane of eukaryotic cells and comprises between 20 to 40 mol% of the membrane^20^. It is a sterol with an essential role in maintaining membrane fluidity and can directly interact with integral membrane proteins^21^. Multiple studies with eukaryotic transporters in mammalian cells have shown that both the human serotonin transporter (hSERT) and human dopamine transporter (hDAT) are modulated in activity following either cholesterol depletion or addition^22^,^23^,^24^. In the case of hSERT, biochemical analyses have shown that cholesterol binding enhances the fraction of the transporter in the outward open confirmation while with the hDAT it stabilises the transporter in an outward open conformation^23^,^25^. Corroborating this, the outward open conformation was observed in the crystal structures of cholesterol or cholesteryl hemisuccinate (CHS) bound *Drosophila melanogaster* dopamine transporter (dDAT) and hSERT^26^,^27^,^28^. The interaction with cholesterol or CHS was increased by mutations of dDAT and hSERT that also decreased the transport kinetic parameters while still retaining functional activity^12^,^27^.

Here we show that cholesterol modulates LAT1 stability and its transporter activity. We provide evidence that cholesterol/CHS interact with LAT1-CD98 and suggest that the LAT1 transporter has two cholesterol/CHS binding sites similar to the binding sites found in the dDAT.

## Results

### Modulation of cholesterol levels alters the LAT1 transport of L-DOPA

**Figure 1:**
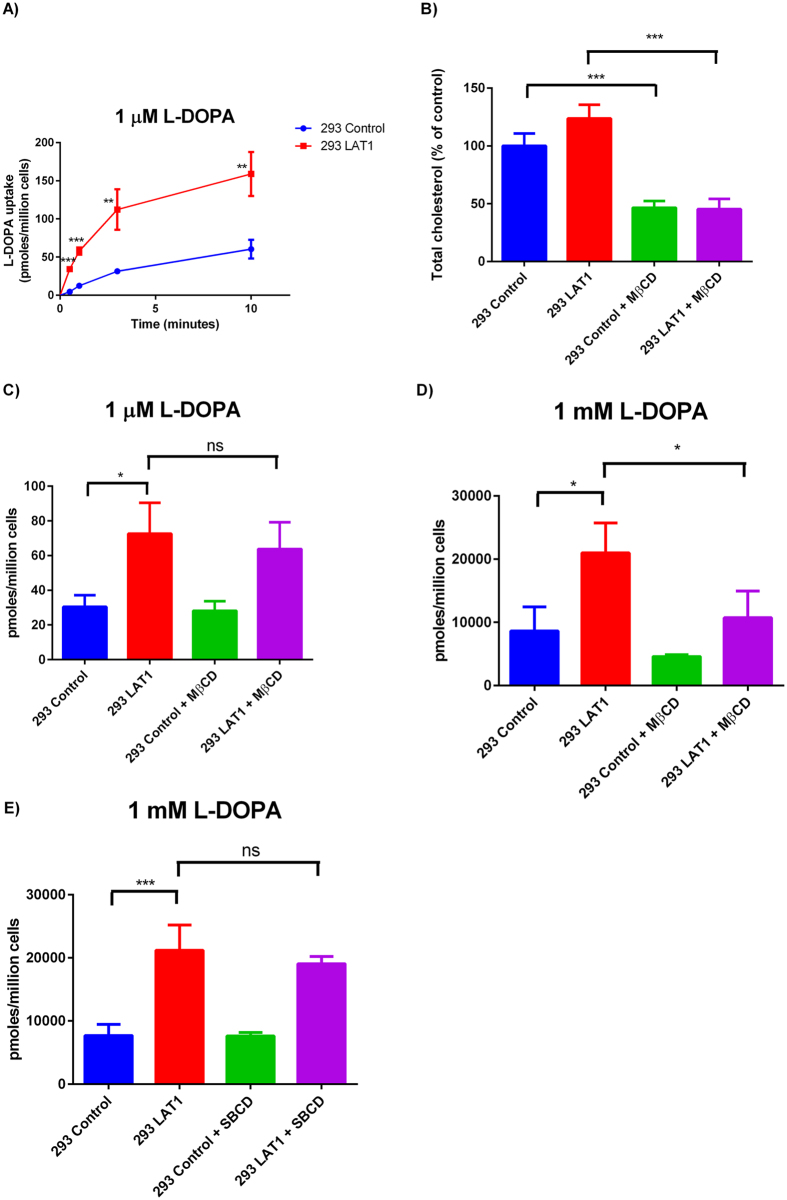
Cholesterol depletion modulates L-DOPA transport in LAT1 overexpressing cells. (**A**) HEK293 control and HEK293 LAT1 cells were incubated over a time course with 1 μM [^3^H]-L-dopa with uptake determined as pmoles per million cells and plotted against time (minutes). HEK293 cells stably transfected with pcDNA3.1 (control) or pcDNA3.1 LAT1 (LAT1) were preincubated with 10 mM methyl β cyclodextrin (MβCD) for 1 hour in serum free medium. (**B**) Total cholesterol (free and ester forms) were determined with a cholesterol quantification assay (fluorometric detection) and are shown relative to untreated control cells. Uptake of 1 μM (**C**) or 1 mM (**D**) [^3^H]-L-dopa was determined at a 3 minute time point in HEK293 control or HEK293 LAT1 cells. (**E**) HEK293 control or HEK293 LAT1 cells were preincubated with 10 mM sulfobutylether-β-cyclodextrin (SBCD) for 1 hour in serum free medium. Uptake of 1 mM [^3^H]-L-dopa was determined at a 3 minute time point in HEK293 control or HEK293 LAT1 cells. Data shown are three independent experiments performed in triplicate and are expressed as mean ± SD (n = 3). Significantly different from control or untreated cells; *(P < 0.05), **(P < 0.01), ***(P < 0.001). No statistical significance is indicated by ns.

A time course for L-DOPA uptake into cells was performed in both HEK293 control cells and stably transfected HEK293 LAT1 cells, in order to validate the use of L-DOPA as a model LAT1 substrate (Fig. 1A). At all time points tested, a significant increase in uptake of L-DOPA was observed in the LAT1 stably transfected cells compared to the control cells. This finding is in agreement with previous studies that have shown L-DOPA to be a substrate of the LAT1 transporter^4^, by an uptake process that is both sodium independent and inhibited by 2-aminobicyclo-[2,2,1]-heptane-2-carboxylic acid (BCH)^29^. L-DOPA was thus used as a model substrate of LAT1 in experiments investigating the role of cholesterol on LAT1 function. To deplete HEK293 cells of cholesterol, the cells were treated for 1 hour with methyl-β-cyclodextrin (MβCD). The relative amount of cholesterol in the cells was determined by a cholesterol quantification assay. There was observed a significant decrease in total cholesterol (cholesterol and cholesteryl ester) in the MβCD treated cells compared to the untreated cells (Fig. 1B). At 1 μM of L-DOPA, no significant difference in L-DOPA up take was detected between treated and untreated HEK293 LAT1 cells (Fig. 1C). However, at 1 mM L-DOPA a significant reduction in L-DOPA uptake was observed in MβCD treated LAT1 cells compared to the untreated LAT1 cells (Fig. 1D). Sulfobutylether-β-cyclodextrin (SBCD), a derivative of cyclodextrin which can interact with cholesterol but not extract it from the plasma membrane^30^, was utilised as a control. SBCD was rationally designed to improve its safety profile *in vivo* and part of this was the removal of the compounds ability to dimerises with itself. As such SBCD is unable to form the 2 to 1 molecular ratio with cholesterol that is required for depletion from the plasma membrane^30^. SBCD treatment was found not to alter the L-DOPA uptake significantly (Fig. 1E) which suggests that the cholesterol depletion effects of MβCD are required for its effect on L-DOPA uptake.

### Cholesterol depletion alters the kinetics of LAT1 mediated transport

**Figure 2:**
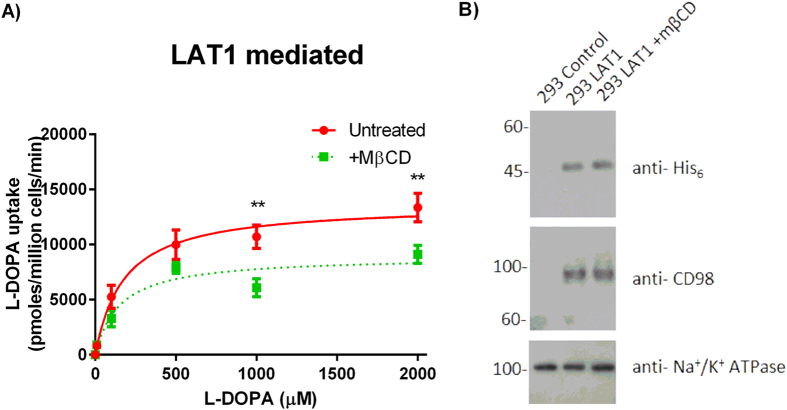
The kinetics of LAT1 mediated transport are altered by cholesterol depletion. (**A**) The LAT1 mediated uptake of L-DOPA was determined ± preincubation with 10 mM MβCD for 1 hour in serum free medium. Following treatment, HEK293 control and HEK293 LAT1 cells were incubated in transport buffer with 1 μM–2000 μM of [^3^H]-L-DOPA. The L-DOPA uptake velocity in the HEK293 control cells was taken away from L-DOPA uptake velocity in the HEK293 LAT1 cells to give a LAT1 mediated transport rate. The concentration of L-DOPA (μM) is plotted against the velocity of LAT1 mediated L-DOPA uptake (pmoles/minute/million cells). Data are expressed as mean ± SD (n = 3). Significantly different from untreated cells; **(P < 0.01). (**B**) Immunoblots for His_6_ tag, CD98 and Na^+^/K^+^ ATPase on cell surface preparations from HEK293 control or HEK293 LAT1 cells ± 10 mM MβCD treatment for 1 hour in serum free medium. Representative western blots are shown and full-length blots are presented in Supplementary Figure 1.

Given the effects of MβCD treatment on the activity of the LAT1 transporter, we investigated the LAT1 mediated kinetics at a range of L-DOPA concentrations at a linear time point (Fig. 2A). The data showed that MβCD treatment altered the kinetics of LAT1 mediated transport (Table 1). The V_max_ was significantly reduced in the treated cells (8506 pmoles/million cells/min) compared to the untreated cells (13925 pmoles/million cells/min), but the K_m_ of L-DOPA uptake between untreated (200 μM) and treated (148 μM) showed no significant difference. This finding could be caused by either alterations of the transport properties of the LAT1 protein or a change in the amount of the transporter at the plasma membrane.

To investigate whether MβCD treatment altered the localisation of the LAT1 transporter, cell surface preparations from untreated and treated cells were immunoblotted. No change in plasma membrane localisation of over expressed LAT1 was observed following MβCD treatment (Fig. 2B). This suggests that the change of uptake kinetics by LAT1 following cholesterol depletion by MβCD is not due to an alteration in the localisation of the transporter complex. From classical Michaelis-Menten kinetics parameters, a change in V_max_ but not K_m_ suggests a non-competitive inhibitor process, more precisely in this case, allosteric modulation^31^.

Figure 2B shows an upregulation of endogenous CD98 in response to increased LAT1 expression in HEK293 cells, an observation initially reported by Khunweeraphong *et al*.^32^,^33^. Correspondingly, the knock out of LAT1 leads to a reduction of CD98 protein levels^32^,^33^. This linkage of CD98 to LAT1 expression levels enables us to utilise cell lines that are only ectopically expressing the LAT1 transporter for protein studies of the LAT1-CD98 heterodimer.

### Detergent solubilised LAT1-CD98 complex is stabilised by a cholesterol analogue (CHS)

**Figure 3:**
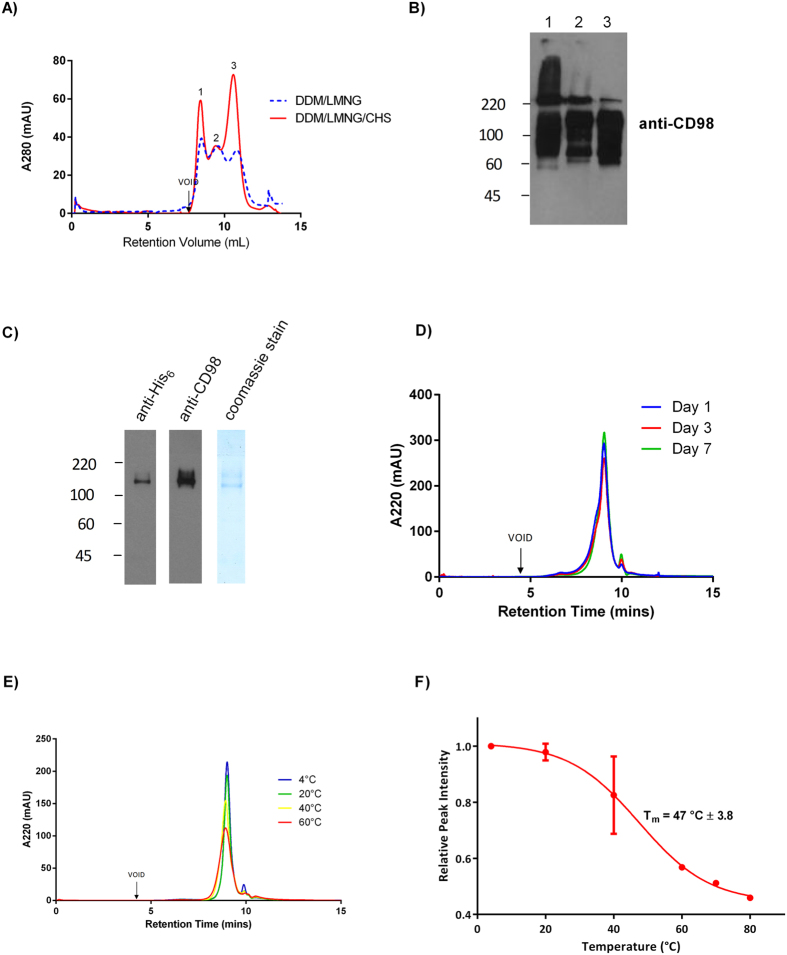
Cholesterol analogue (CHS) stabilises LAT1-CD98 during affinity purification. (**A**) LAT1 was purified by immunoaffinity precipitation utilising the V5 epitope from HEK293S GnTIˉ cells stably expressing pcDNA3.1 LAT1-V5-6xHis. Representative SEC profiles detected by absorbance at 280 nm are shown. (**B**) The peaks eluted at 8.4 mL (1), 9.5 ml (2) and 10.5 ml (3) from the SEC profile with CHS were analysed by immunoblotting for CD98 in non-reducing conditions. (**C**) Peak 3 from SEC was concentrated, run on SDS-PAGE then coomassie stained or immunoblotted for the His_6_ tag and CD98 in non-reducing conditions. (**D**) Stability of the purified LAT1-CD98 at 4 °C was monitored for up to 7 days after purification by analytical SEC. (**E**) The purified LAT1-CD98 was heated for 10 minutes at the indicated temperatures and run on analytical SEC. (**F**) The effect of the heat stress on the purified LAT1-CD98 was quantified from the chromatograms by calculating the normalised absorbance (n = 3). The normalised absorbance of the purified LAT1-CD98 was plotted against temperature with the melting point determined (T_m_ ± standard error).

To investigate if cholesterol interacts with human LAT1 directly, ectopically expressed protein from HEK293 GnTI^-^ cells was solubilised in detergents with and without the water-soluble cholesterol analogue CHS before immunoaffinity purification of LAT1-CD98. Distinct size-exclusion chromatography (SEC) profiles were observed with and without CHS (Fig. 3A). Peaks 1 and 3 had increased peak heights in presence of CHS. Additionally, peak 3 with CHS had a shifted retention time compared with peak 3 in the purification without CHS. Each of the three peaks from the purification with CHS were immunoblotted for CD98 in non-reducing conditions and an over exposed immunoblot is shown (Fig. 3B). Despite the denaturing conditions of SDS-PAGE, a smear above 220 kDA was observed in peaks 1 and 2 that was not present in peak 3. This is suggestive of aggregation of the purified proteins in peaks 1 and 2, thus all following experiments utilised peak 3 from purifications that contained CHS. Peak 3 was immunoblotted in non-reducing conditions for CD98 and His_6_ tagged LAT1. Both proteins of the heterodimer were detected and had the characteristic smear of a glycosylated protein (Fig. 3C). The immunoblots and coomassie stained SDS-PAGE gel reveal a band consistent with the 123 kDa molecular weight of the heterodimer (Fig. 3C). In order to further biochemically characterise the purified complex in near to native conditions, the complex was run on analytical SEC. A monodisperse peak corresponding to the LAT1-CD98 heterodimer was observed on day 1 (Fig. 3D). The SEC analysis was repeated 3 and 7 days after purification in order to determine stability of the purified LAT1-CD98 stored at 4 °C over time. The complex was kinetically stable in the SEC buffer for 3 and 7 days at 4 °C with no detectable aggregation or denaturation by analytical SEC (Fig. 3D). Taken together, the data from the immunoblots and chromatographic profiles produced from the analysis of peak 3, it can be concluded that CHS is necessary for successful purification of the LAT1-CD98 heterodimer.

To determine the thermal stability of the LAT1-CD98 complex, samples in SEC buffer were heated to temperatures between 4–100 °C and analysed by HPLC-SEC. The peak height and monodispersity of the LAT1-CD98 samples decreased with increasing temperature (Fig. 3E). To quantify this, the normalised absorbance relative to the protein sample at 4 °C was plotted against temperature. A dose response curve to heating was obtained and the thermal stability of the LAT1-CD98 in SEC buffer was defined with the T_m_ found to be 47 °C (Fig. 3F).

### CHS stabilised LAT1-CD98 complex interacts with leucine

**Figure 4:**
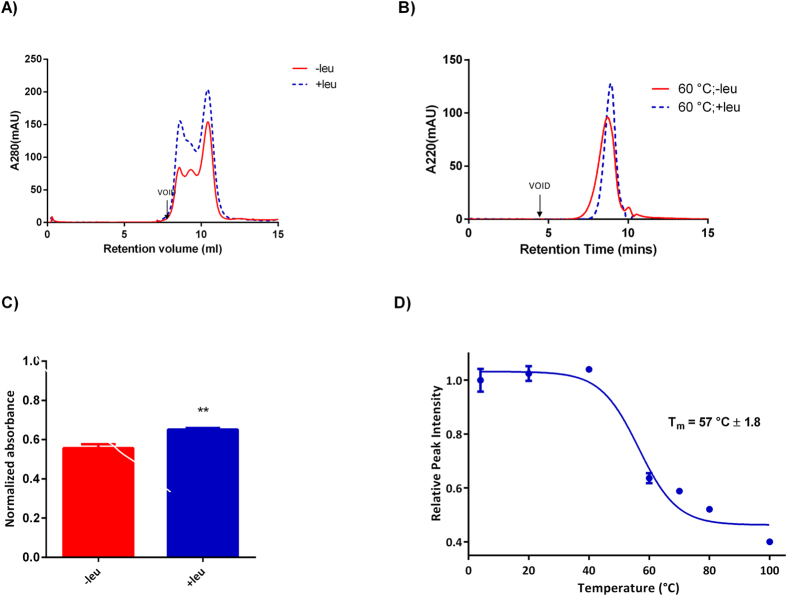
The effect of leucine on purification and thermal stability of LAT1-CD98. (**A**) SEC profiles of protein purification performed in the presence or absence of 50 mM leucine. (**B**) The effect of presence or absence of 50 mM leucine on the thermal stability of LAT1-CD98 was determined by heating stressing, at 60 °C for 10 minutes followed by HPLC-SEC analysis. (**C**) The effect of the heat stress on the purified LAT1-CD98, in the absence or presence of leucine was quantified by calculating the normalised absorbance (n = 3). Significant difference from purified LAT1-CD98 without leucine; **(P < 0.01). (**D**) The effect of the heat stress on the purified LAT1-CD98 with leucine was quantified from the analytical SEC profiles by calculating the relative peak intensity and normalised to the intensity at 4 °C (n = 3). The normalised absorbance for the peak intensity of the purified LAT1-CD98 with leucine was plotted against temperature with the melting point determined (T_m_ ± standard error).

Ligand studies were undertaken to determine the thermal and conformational stability of the purified LAT1-CD98, as an interaction with ligand can be an indirect indicator that the protein is correctly folded. Leucine was chosen for this purpose, as it absorbs ultraviolet light at 280 and 220 nm negligibly, and is stable in solution during the experimental time frame. The addition of leucine resulted in a 39% increase in the peak height at 10.4 mL (peak 3) in the SEC profile (Fig. 4A). To determine whether this increase was due to increased stability of LAT1-CD98, samples of LAT1-CD98 purified in the presence and absence of leucine were heat stressed at 60 °C for 10 mins and then analysed by HPLC-SEC (Fig. 4B). This showed a significantly decreased monodispersity in the absence of leucine and a 9% higher normalised absorbance in the presence of leucine, indicative of a thermal stabilising effect (Fig. 4C). The melting curve for LAT1-CD98 in the presence of leucine showed that the T_m_ increased to 57 °C (Fig. 4D) compared to 47 °C without leucine. This suggests that not only is CHS required for successful protein purification but that the purified protein retains its ability to interact with substrate compounds. However, it should be noted that this assay does not distinguish between specific and nonspecific interactions of leucine with the LAT1-CD98 heterodimer.

### Conservation of cholesterol binding domains

**Figure 5:**
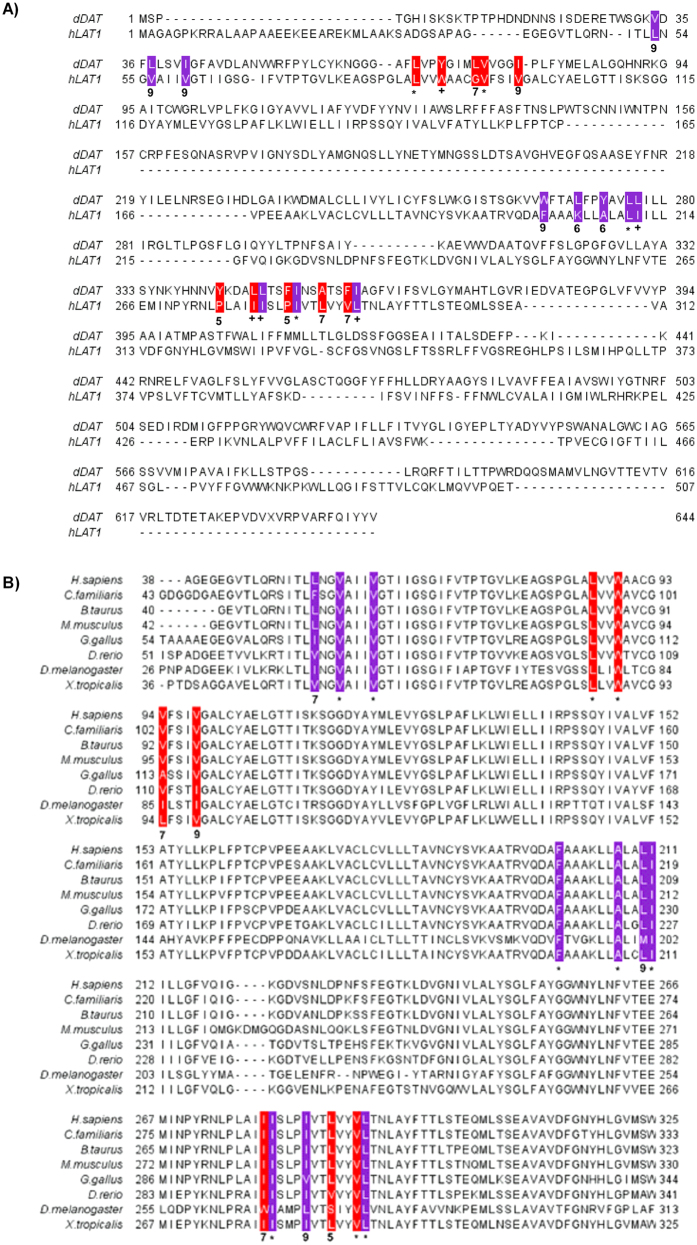
Putative conservation of the cholesterol binding sites of the *drosophila melanogaster* dopamine transporter in the human LAT1 transporter. (**A**) Sequence alignment of the cholesterol/CHS binding sites I (purple) and site II (red) from dDAT and human LAT1. Identical residues are scored with an asterix (*), equivalent residues with a plus (+) and similar residues scored on a scale from 1–9, with 1 being variable and 9 being the maximal level of similarity. (**B**) Sequence comparison of the amino acid residues of site 1 and site 2 of the cholesterol binding sites of human LAT1 to orthologues. A conservation is given below each residue.

**Figure 6:**
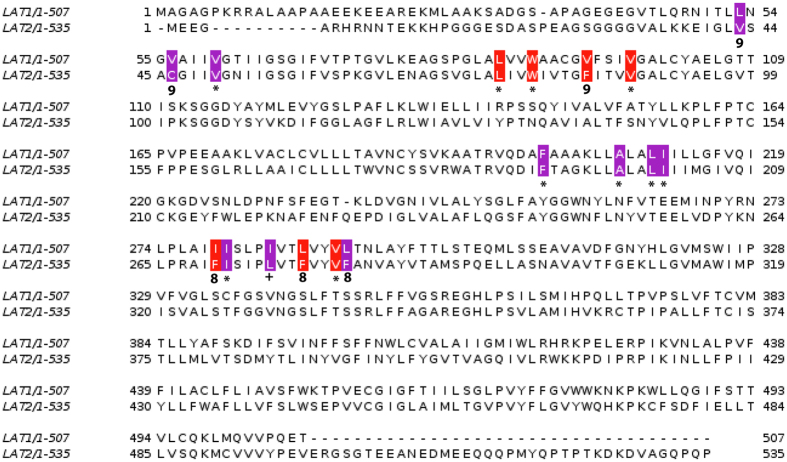
Comparison of the putative cholesterol binding site residues of LAT1 versus the LAT2 transporter sequence. Sequence alignment of the putative cholesterol/CHS binding sites I (purple) and site II (red) from human LAT1 and LAT2. Identical residues are scored with an asterix (*), equivalent residues with a plus (+) and similar residues scored on a scale from 1–9, with 1 being variable and 9 being the maximal level of similarity.

**Figure 7:**
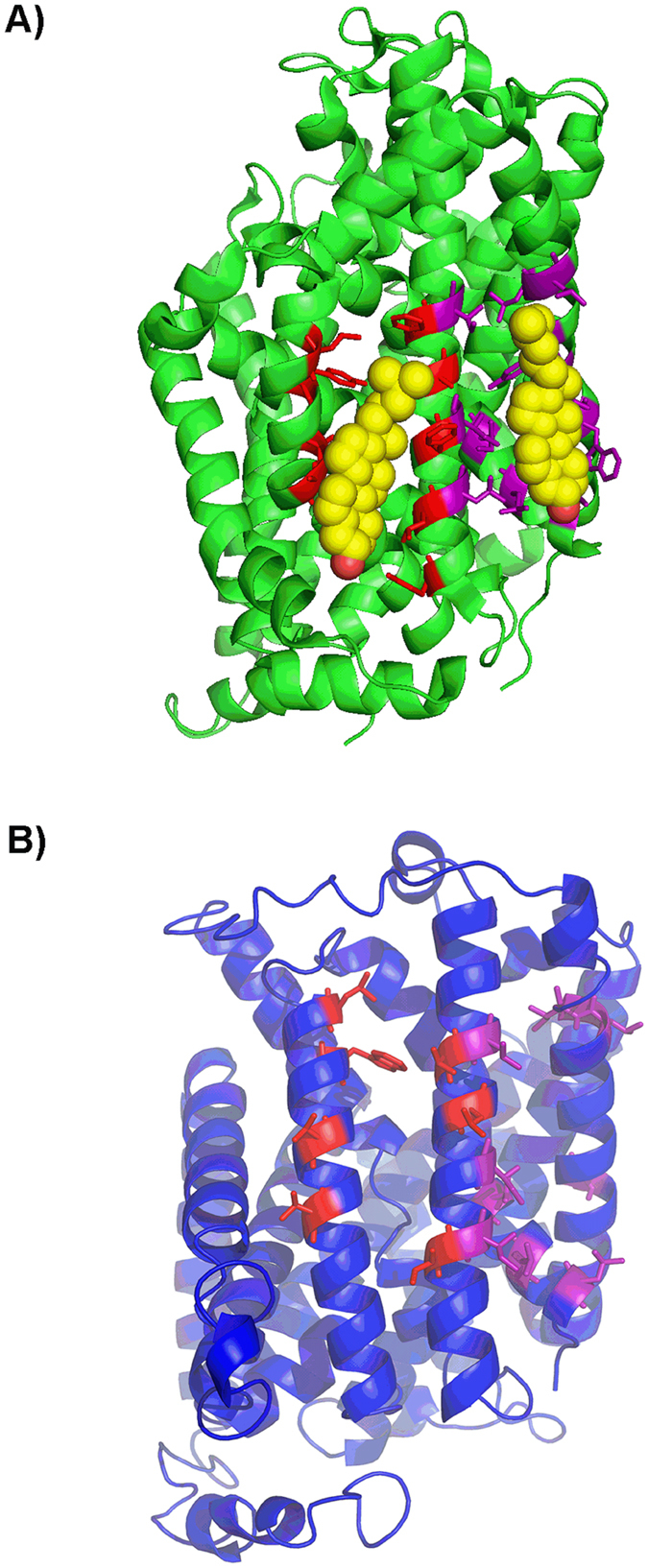
Location of the putative cholesterol binding sites on the predictive 3D model of LAT1. (**A**) Crystal structure of dDAT (PDB: 4xpf) bound to cholesterol (yellow and red spheres) (**B**) Predictive 3D structure of LAT1 generated by the I-TASSER server and optimised to improve backbone stereochemistry. Key residues in the cholesterol/CHS binding sites I (purple) and II (red) of dDAT and the corresponding residues in the putative sites of LAT1 are shown as sticks.

To build on the experiments with cells and purified protein we investigated if the cholesterol/CHS binding sites of the dDAT are conserved in LAT1 by *in silico* methodologies. An alignment of LAT1 and dDAT was performed using PROMALS3D, which allows for the alignment of distantly related sequences by taking into account structural information, predicted or otherwise^34^. The two transporters have a low sequence identity of 19.6% but are predicted to share the same LeuT structural fold. Most of the cholesterol interacting residues in binding sites I (91%) and II (70%) of dDAT are identical, equivalent or have similar physico-chemical properties (conservation value > 5) to corresponding residues in LAT1 (Fig. 5A). To test whether these residues may have functional importance for the LAT1 transporter and are thereby conserved during evolution, we performed a multiple sequence alignment of LAT1 orthologues from a diverse group of 8 metazoans. Ten residues were identical and seven similar out of 17 residues, across both binding sites in the 8 orthologues (Fig. 5B). An alignment of LAT1 and LAT2 (Fig. 6) found residues comprising both of the cholesterol/CHS putative binding sites were conserved, with all residues having a conservation scores >7. The conserved residues were located on adjacent helices in the predicted 3D structure of LAT1 (Fig. 7B), which is consistent with the requirement for the cholesterol interacting residues in the binding sites to be in close proximity, as seen in the dDAT structures (Fig. 7A)^26^,^27^.

## Discussion

LAT1 was first cloned in 1998, found to interact with CD98 via a disulphide bond and is the transport component of the heterodimer^1^,^2^,^17^,^35^. To date our mechanistic knowledge of how LAT1 functions and interacts with substrates is derived from *in silico* models generated using low sequence similarity homologues from prokaryotes that have the LeuT fold^14^,^15^. These studies have been successful in identifying novel ligands and suggesting that the transporter acts by the alternative access mechanism^17^. However, to our knowledge, there are no experimental or modelling studies in the literature concerning the LAT1 transporter that take into account structural knowledge gained from eukaryotic transporters with the conserved LeuT fold. As a result, structural insights to LAT1 function resulting from its eukaryotic origin, have been missed. This is the first study to both model and test this experimentally for the human LAT1 transporter.

Two well-characterised eukaryotic transporters, that have the LeuT fold, are the serotonin and dopamine transporters. Both have established interactions with cholesterol, and so we tested to see if LAT1 also has this feature. We found, following acute cholesterol depletion that LAT1 mediated kinetics of L-DOPA uptake were altered, with a decrease in V_max_ but no change in the K_m_. This is consistent with a reduced maximal transport activity but without discernible change in substrate affinity. The depletion of cholesterol has been found to also result in reduced activity for the hDAT^24^ which led us to further investigate the mechanism of this modulation, to determine whether it is common between the two transporters.

Biochemical studies have suggested that cholesterol can either stabilise or induce the outward conformation for the hDAT and hSERT^23^,^25^. In the crystal structures of dDAT and hSERT, both transporters are bound to cholesterol and or CHS in the outward open conformation^26^,^27^,^28^. To determine whether the modulation of LAT1 activity we observed was mediated indirectly by changes in membrane fluidity, for example, or whether it was through a direct interaction, LAT1 was purified. We found that the LAT1-CD98 heterodimer in the presence of CHS was stable and was further stabilised by a ligand, suggesting the complex was natively folded. In contrast, the heterodimeric complex could not be purified in the absence of CHS. The HPLC-SEC based thermostability assay utilised here, is a similar approach to that used for the GABA receptor to determine the binding constants of known ligands and identify a novel agonist for this receptor^36^. This approach may prove useful in establishing interactions of LAT1-CD98 with known LAT1 ligands and novel compounds, with the intention of inhibiting LAT1 for cancer treatment or to enhance brain penetration of compounds^37^,^38^.

Extensive work on GPCRs has revealed two mechanisms by which CHS potentially stabilises membrane proteins, namely through the modulation of the geometry of detergent micelles or through direct interaction with the protein^39^. CHS is required for purification of a stable LAT1-CD98 complex suggesting either or both of the mechanisms above are relevant. A sequence comparison of dDAT and LAT1 reveals conservation of residues that could form two putative cholesterol/CHS binding sites. The conservation of these sites across LAT1 orthologues lends credence to the functional importance of these putative binding sites. Taken together, we conclude from these data that cholesterol modulates the activity of LAT1, and does so most likely through a direct interaction.

For some membrane proteins, a cholesterol/CHS interaction has been proposed to occur through cholesterol interacting domains referred to as CRAC/CARC domains^40^. CRAC and CRAC-like motifs, are very different when compared with the putative cholesterol/CHS binding sites identified in LAT1 through homology with dDAT. Unlike the CRAC motif, in which the residues essential for cholesterol interaction are contiguous, the cholesterol/CHS interacting residues of dDAT are separated in sequence space and can be proximal in 3D space^26^. Annotation of our predictive model of the LAT1 structure shows that the residues of the putative binding sites are on adjacent transmembrane helices which is similar in arrangement to dDAT. Furthermore, the recently solved structure of hSERT^28^ found CHS bound through a motif that conforms neither to the CRAC motif nor the two dDAT binding sites.

When LAT1 was overexpressed in the HEK293 cells we found that CD98 was upregulated. This has been noted by a number of previous studies and occurs by an unknown mechanism^32^,^33^,^41^. Our study found no evidence to suggest that LAT1 exists as a monomer in mammalian cells as the three fractions isolated and tested from the purification on a western blot all contained CD98. This shows that a lack of CD98 was not the reason for the instability found in SEC peaks 1 and 2. This is an important aspect to consider as LAT2 shares 52% amino acid identify with LAT1 and has been shown to be stabilised by its interaction with CD98^19^. A report concerning the LAT2-CD98 complex found that the addition of lauryl maltose neopentyl glycol and CHS during the purification stabilised the complex by an unknown mechanism^42^. However, the individual importance or otherwise of the LMNG and CHS was not individually tested^42^. Given the conservation between LAT1 and LAT2, it would be interesting to determine the effects of CHS on thermal and kinetic stability of LAT2 and of cholesterol depletion in cells on the kinetics of LAT2 uptake.

The closest LAT1 homologue in prokaryotes is the antiporter for serine/threonine SteT that has had mechanistic information determined through the use of single molecule force spectroscopy^43^. This has found that ligand binding enhanced the kinetic stability and increased the flexibility of the transporter compared to the unbound form. In our study the addition of leucine enhanced the thermostability of the LAT1-CD98 complex but how this effects the rigidity of the complex is unknown. This is difficult to predict for the unbound or substrate bound forms of LAT1-CD98 due to the additional complexity of the heterodimeric nature of the transporter compared to SteT.

In this present study we have taken advantage of the cholesterol interacting properties of cyclodextrin derivatives. Methyl-β-cyclodextrin forms dimers each of which interacts with a cholesterol molecule, extracting it from the plasma membrane^44^. Sulfobutylether-β-cyclodextrin is unable to homodimerise and as a result does not remove cholesterol from the plasma membrane. In our experiments the transport of L-DOPA in LAT1 transfected cells was unaffected by incubation with sulfobutylether-β-cyclodextrin. Given the hydrophilic succinate moiety of the CHS, we presumed that methyl-β-cyclodextrin was unsuitable for depletion of CHS from the LAT1-CD98 complex purified in the presence of CHS. Cyclodextrins have a number of proposed pharmacological uses. A cyclodextrin derivative has recently been proposed as a treatment option to promote atherosclerosis regression by removal of plaques^45^ and they are used as pharmacology excipients. In the current study we have not investigated *in vivo* but it would be interesting to do so in the future due to the potential effect on nutrient transport of these cyclodextrin derivatives utilised in treatment regimens.

In summary, we have been able to address some fundamental questions involving LAT1-CD98 interaction with cholesterol and how this affects stability and kinetic values. This should facilitate structural studies of LAT1 that will ultimately be able to define how the transport cycle progresses at atomic/chemical level and the role of cholesterol in these processes.

## Methods

### Materials

Tritium labelled L-DOPA was acquired from Moravek (California, USA) with a specific activity of 3.6 Ci/mmol. The V5 resin and V5 peptide were obtained from Biotool (Houston, USA). Cholesteryl hemisuccinate tris (CHS) and n-dodecyl beta maltoside (DDM) detergents were purchased from Generon (Maidenhead, UK). All other regents and chemicals, unless otherwise stated, were purchased from Sigma (Poole, Dorset, UK).

### Cell Culture

HEK293 (ATCC, Middlesex, UK) and HEK293S GnTIˉ (HEK293SG, ATCC), were cultured adherently in high glucose Dulbecco’s Minimal Essential Medium (DMEM) supplemented with 10% v/v Foetal Bovine Serum (FBS). The cells were incubated at 37 °C with 5% CO_2_ and passaged every two to three days. HEK293 pcDNA3.1 (control) and HEK293 pcDNA3.1 LAT1-V5-His_6_ (LAT1) stable cell lines were previously generated by lipofectamine 2000 transfection, G418 treatment and single cell cloning^3^. Suspension cultures of HEK293S GnTIˉ cells stably overexpressing LAT1 were seeded from adherent cultures and maintained in either 1 or 1.5 L of media in spinner flasks at 150 rpm. The suspension culture medium was composed of MEM Joklik modification with the addition of 13.4 mM glucose, 24 mM sodium bicarbonate, 10% w/v primatone, 1% w/v pluronic and 5% v/v FBS. Harvesting of the suspension cultures was done every four days for the HEK293S GnTIˉ LAT1 cells.

### MβCD treatment, cholesterol quantification and cell surface preparation

To deplete cholesterol, the HEK293 cells were treated with serum free DMEM ± 10 mM methyl-β-cyclodextrin (MβCD) for 1 hour at 37 °C with 5% CO_2_. Cells were used for drug uptake assay, cell surface preparation or total cholesterol quantification (cholesterol/cholesteryl ester).

The cholesterol quantification assay (ab65359, Abcam, Cambridge, UK) was used with fluorometric parameters according to manufacturer’s protocol. In brief, lipids were extracted from cells with chloroform: isopropanol: NP-40 (7:11:0.1), pelleted and organic phase air dried. Assay was performed in the presence of cholesterol esterase with florescence measured on a microplate reader with 535 nm excitation and 595 nm emission filters.

To isolate cell surface proteins, a cell surface protein isolation kit, (Thermo Scientific) based on biotinylation and affinity binding to NeutrAvidin resin was used as previously described^46^.

### Drug uptake

Functional drug uptake assays were performed using tritium labelled levodopa (^3^[H]-L-DOPA) as a tracer at 0.15 μCi/mL in transport medium (0.01 μM to 2 mM unlabelled L-DOPA; 25 mM HEPES, pH 7.4; Hank’s buffered saline solution (HBBS); 0.1% w/v BSA). The various HEK293 cell-lines were seeded 24 hours before the assay. Medium was aspirated and cells washed with HBBS, before transport medium, warmed to 37 °C was added. At the end of the assay, the transport medium was aspirated off and transport stopped by washing cells with ice cold HBBS three times. Cells were lysed by incubating at 37 °C in 5% w/v SDS for 30 minutes. The amount of radiation in the lysates was measured by liquid scintillation in disintegrations per minute which were used to calculate the amount of L-DOPA taken up as pmoles/million cells.

### Immunoaffinity purification

HEK293S GnTIˉ LAT1 cell pellets were retrieved from cryostorage and thawed on ice in lysis buffer (10% v/v glycerol; Dulbecco’s phosphate buffered saline, pH 7; protease inhibitor tablets (Pierce)). Thawed cells were lysed with a TissueRuptor (Qiagen) followed by sonication. Cell nuclei and debris were removed by centrifugation at 23,500 *g* for 20 minutes. The supernatant was then ultracentrifuged at 100,000 *g* for 1.5 hrs to isolate the membrane fraction from the soluble cytosolic fraction. The membranes were suspended by dounce homogeniser for solubilisation in TBS1 (1.5% w/v DDM, 20 mM Tris-Cl, 300 mM NaCl, and 10% w/v glycerol at pH 8 in the absence or presence of CHS). Solubilisation was performed overnight, concomitantly with V5 affinity gel incubation. The fraction of the solubilisation suspension not bound to the affinity gel, was removed by decanting the supernatant after centrifugation at 1500 *g* for 2 minutes. Resin was then washed in SEC buffer with DDM above CMC, 100 mM Tris-Cl, 300 mM NaCl, and 10% w/v glycerol at pH 8 in the absence or presence of CHS. LAT1-CD98 was eluted by incubating the washed resin with V5 peptide. Further purification was performed by size exclusion chromatography on a Superdex 200 10/300 column (GE Healthcare). The purified protein was concentrated using a 100 kDa cut-off polyethersulfone centrifugal filter and the pure protein concentration calculated as follows; A_280_/ε with ε = 1.331, calculated from combined primary sequence of LAT1 and CD98 by ExPASy ProtParam^47^.

### Immunoblotting

Protein samples were incubated at room temperature in SDS loading buffer (0.5 M Tris-Cl; 30% v/v glycerol; 10% v/v SDS; 0.012% w/v bromophenol blue) before loading on to 12.5% Tris-glycine SDS-PAGE gels. Dithiothreitol or β-mercaptoethanol was included in the loading buffer when reducing conditions were required. 20–40 μg total protein was loaded per well for cell-lysates and 5 μL from analysis of LAT1-CD98hc purifications. Gels were run at 200 V for 1 hr and proteins transferred from gel to PVDF membranes by wet blotting at 100 V for 1.5 hrs. The membranes were blocked in 5% w/v semi-skimmed milk. Immunoblots were incubated with anti-His_6_ mouse monoclonal antibody (1:1000; Abcam) or anti-CD98 rabbit polyclonal antibody (1:1000; H300 Santa Cruz) or α 1 sodium potassium ATPase mouse monoclonal antibody (1:2000, clone 464.6 Abcam). Visualisation was done using anti-mouse or anti-rabbit HRP conjugated secondary antibodies by chemiluminescence.

### Thermostability experiments

LAT1-CD98 was purified in SEC buffer and the fractions corresponding to the heterodimer were pooled, concentrated and analysed by HPLC-SEC. To determine the thermal stability of LAT1-CD98, the protein in the desired buffer conditions, was incubated at a range of temperatures for 10 minutes. 3 to 5 μg of protein from each sample were injected and analysed using the BIO-SEC5 HPLC column (Agilent). The peak height at A_220_ was normalised by dividing by the peak area and expressed as a fraction of the normalised peak height at 4 °C. This is referred to as the normalised absorbance. Normalised absorbance was plotted against temperature and the data analysed by a non-linear regression to the Boltzmann sigmoidal function using GraphPad Prism 6 (GraphPad Software, Inc., La Jolla, USA).

### *In silico* protein structure modelling and conservation analysis

The amino acid sequences for human LAT1 and the *Drosophila melanogaster* dopamine transporter (dDAT) (NCBI accession numbers NP_003477.4 and NP_523763.2 respectively) were aligned using PROMALS3D^34^. The alignment was used to identify residues in cholesterol binding sites I & II of the dDAT that were conserved in LAT1, thus defining putative cholesterol binding sites in LAT1. An alignment of LAT1 orthologous sequences and LAT2 (Uniprot idenitfier:Q9UHI5-1) was performed using Clustal O^48^ to determine whether the putative cholesterol binding sites are conserved between orthologues. Orthologues were chosen from *Canis lupus familiaris, Bos Taurus*, Rattus norvegicus, *Mus musculus, Gallus gallus, Danio rerio, Drosophila melanogaster*, and *Xenopus tropicalis.* (NCBI accession numbers XP_850176.2, NP_777038.1, NP_059049.1, NP_035534.2, NP_001025750.1, NP_001121830.1, NP_001245996.1, and NP_001135465.1 respectively). Annotation and scoring of conservation at each position was done in Jalview^49^, residues in binding site I & II were annotated in purple and red respectively. A model of LAT1 was generated in order to determine the proximity in space, of the residues in the putative binding sites. The best I-TASSER server^50^ generated 3D model of LAT1 was optimized using Modrefiner^51^ to improve backbone stereochemistry as assessed by Ramachandran plot analysis using RAMPAGE. Residues in putative binding sites were annotated on the resulting predicted structure in purple and red for sites I & II respectively.

### Statistical tests & kinetic calculations

All statistical tests were performed with GraphPad Prism 6. For two different conditions, a two tailed t-tests was performed while for multiple comparisons an ANOVA with post hoc Tukey’s test was carried out.

Kinetics of L-DOPA uptake were determined by selecting a time point when linear transport was occurring and then subtracting the drug accumulation in HEK293 control cells from drug accumulation in HEK293 LAT1 cells. This provides the LAT1 mediated fraction. V_max_ was calculated by plotting the rate of drug transport by LAT1 (pmoles/min/million cells) against L-DOPA concentration (μM). GraphPad Prism 6 was used to calculate Michaelis–Menten values for LAT1 mediated L-DOPA uptake.

## Additional Information

**How to cite this article:** Dickens, D. *et al*. Modulation of LAT1 (SLC7A5) transporter activity and stability by membrane cholesterol. *Sci. Rep.* **7**, 43580; doi: 10.1038/srep43580 (2017).

**Publisher's note:** Springer Nature remains neutral with regard to jurisdictional claims in published maps and institutional affiliations.

## Supplementary Material

Supplementary Figure 1

## Figures and Tables

**Table 1:** Kinetics of LAT1 mediated transport ± MβCD treatment for the uptake of L-DOPA.

	Untreated	+MβCD
**V**_**max**_ (pmoles/million cells/min)	13925 (923)	8506 (684)*
**K**_**m**_ (μM)	200 (78)	148 (22)

Significantly different from untreated LAT1 mediated transport of L-DOPA *(P < 0.05). n = 3 with standard deviation in brackets.
