# Supplementary material for: Modulation of LAT1 (SLC7A5) transporter activity and stability by membrane cholesterol
Source: Sci Rep. 2017 Mar 8;7:43580. doi: 10.1038/srep43580 (PMC5341093; doi:10.1038/srep43580)

Supplementary Figure 1: Original full-length western blots used for Figure 2B.

Modulation of LAT1 (SLC7A5) transporter activity and stability by membrane cholesterol

David Dickens[1^*], George N. Chiduza[2^], Gareth S. A. Wright[2], Munir Pirmohamed[1], Svetlana V. Antonyuk[2], and S. Samar Hasnain[2*]

^=equal contribution

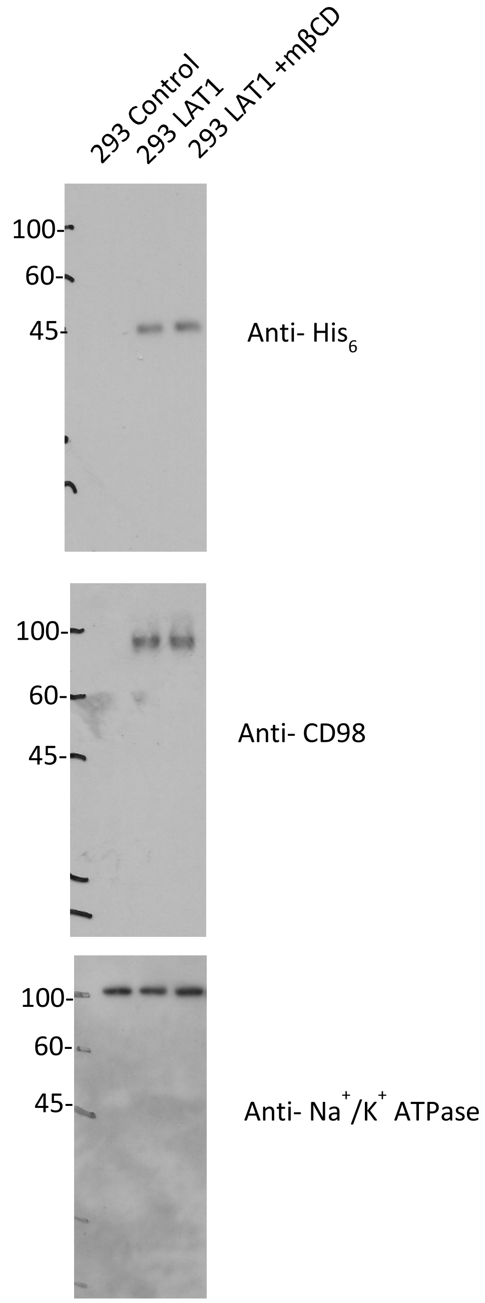

Supplement: Supplementary Figure 1 [file srep43580-s1.pdf]
